# Complete Genome Sequences of *Enterobacter cancerogenus* CR-Eb1 and *Enterococcus* sp. Strain CR-Ec1, Isolated from the Larval Gut of the Greater Wax Moth, *Galleria mellonella*

**DOI:** 10.1128/genomeA.00044-18

**Published:** 2018-02-15

**Authors:** Joon-hui Chung, Haeyoung Jeong, Choong-Min Ryu

**Affiliations:** a The Agricultural Genome Center, National Institute of Agricultural Sciences, Rural Development Administration (RDA), Jeonju, Republic of Korea; b Infectious Disease Research Center, Korea Research Institute of Bioscience and Biotechnology (KRIBB), Daejeon, Republic of Korea; c Department of Biosystems and Bioengineering Program, Department of Biosystems and Bioengineering, KRIBB School of Biotechnology, Korea University of Science and Technology (UST), Daejeon, Republic of Korea

## Abstract

*Enterobacter cancerogenus* CR-Eb1 and *Enterococcus* sp. CR-Ec1 were isolated from the larval gut of *Galleria mellonella*, the greater wax moth. Here, we report the completed and annotated genome sequences of insect gut-dwelling bacteria.

## GENOME ANNOUNCEMENT

The larvae of the greater wax moth (*Galleria mellonella* L.) have been used as a model animal organism for studying the pathogenicity and host-gut microbiome interaction (1, 2). Recent data suggest that the gut bacteria from *G. mellonella* confers wax degradation (3). To investigate the dynamics of gut microbiota and identify potential gut bacteria responsible for wax degradation, we isolated culturable bacteria from the larval gut. Third- to fourth-instar *G. mellonella* larvae were purchased from S-WORM (Cheonan, Republic of Korea). The larvae were fed an artificial diet (600 g rice bran, 600 g wheat bran, 4.5 g yeast extract, 2 g CaCO_3_, 250 ml glycerol, 600 ml honey, 600 mg vitamin B complex, and 175 ml distilled water) at 37°C (4). The samples from one larval gut were macerated with glass beads and phosphate-buffered saline buffer and streaked onto 10-fold diluted tryptic soybean agar. *Enterobacter cancerogenus* CR-Eb1 and *Enterococcus* sp. CR-Ec1 were isolated and identified from the larval gut samples. *E. cancerogenus* (syn. *E. taylorae*) and *Enterococcus* spp. have been reported to be opportunistic human pathogens that infect the urinary tract and open wounds (5–9).

Genome sequencing was performed on a PacBio RS II platform using P6-C4 chemistry, with one single-molecule real-time (SMRT) cell per sample, at the National Instrumentation Center for Environmental Management, Seoul National University (Seoul, Republic of Korea). Sequencing coverages for CR-Eb1 and CR-Ec1 were 90.7-fold and 222.9-fold, respectively. Genome assemblies obtained with the RS_HGAP_Assembly.2 protocol under SMRT Analysis version 2.3.0 (Pacific Biosciences, Menlo Park, CA, USA), followed by circularization using Circlator (10), were further corrected by running two successive rounds of the RS_Resequencing.1 protocol. The CR-Eb1 genome has a 4,796,512-bp chromosome (55.78% G+C content), while CR-Ec1 has a 3,819,143-bp chromosome (42.4% G+C content) and a 70,706-bp plasmid (36.48% G+C content), where all replicons have circular structure. CR-Eb1 was classified as *E. cancerogenus* on the basis of the average nucleotide identity by orthology (OrthoANI) algorithm (99.88%) (11) and the Genome-to-Genome Distance Calculator (90.80% of DDH estimate; https://ggdc.dsmz.de/distcalc2.php) using the genome sequence of type strain ATCC 33241 (GCA_900185905) as the reference. CR-Ec1, however, could not be assigned species-level taxonomy because the analyzed values were below the cutoff, *Enterococcus casseliflavus* ATCC 49996^T^ (GCA_000393915) being the closest strain.

Genome sequences were annotated using the NCBI Prokaryotic Genome Annotation Pipeline (https://www.ncbi.nlm.nih.gov/genome/annotation_prok) and the Rapid Annotations using Subsystems Technology (RAST) server (12). Island Viewer (13) analysis identified no virulence factors or antimicrobial resistance genes in either genome, and antiSMASH (14) predicted biosynthetic gene clusters for enterobactin (CR-Eb1) and dehydrosqualene (CR-Ec1). In conclusion, the complete genome sequences of these two bacterial isolates will provide insights into the infection and control of microbiota by host and/or dietary factors.

### Accession number(s).

The complete genome sequences have been deposited in DDBJ/ENA/GenBank under the accession numbers CP025225 (*E. cancerogenus* CR-Eb1) and CP025223 and CP025224 (*Enterococcus* sp. CR-Ec1).
